# Practical Notes and Formulæ

**Published:** 1886-01-20

**Authors:** 


### PRACTICAL NOTES AND FORMULÆ.

**Sciatica.**—Dr. James E. Wilson (New England Medical Monthly) recommends—

| | | |
|---|---|---|
| ℞. | Chloral hydrat | 3 dr. |
| | Bromide of soda | 3 dr. |
| | Morphia sulph | 6 gr. |
| | Quinia sulph | 54 gr. |
| | Pulverized camphor | ½ dr. |
| | Elixir taraxicum comp | 6 ozs. |
| | Tincture aconite | 24 drops. |

M. Sig. A desertspoonful every three hours until relieved.

**Acute Pleurisy.**—Professor Da Costa (Peoria Medical Monthly) often orders—

| | | |
|---|---|---|
| ℞. | Tincture aconiti rad | 30 min. |
| | Potassi acetatis | ½ oz. |
| | Morphia sulphatis | ½ gr. |
| | Liquid potassi citratis | 2 ozs. |
| | Syrupi tolu | 1 oz. M. |

Sig. Two teaspoonfuls every three hours.

**Dyspepsia.**—Dr. J. H. Bundy says: If you have a case of indisposition in which your patient throws up everything taken, give:

| | | |
|---|---|---|
| ℞. | Fluid extract cascara | 1 oz. |
| | Extract of malt | 2 ozs. |
| | Fluid extract berberis aquifolium | 1 oz. |
| | Acid hydrocyanic dil | 1 dr. M. |

Sig. Teaspoonful directly after meals, or oftener if there is pain or distress, with belching of gas or wind from the stomach. If, with any of the above symptoms, there seems to be a sluggish liver, give nux vomica in proper quantity.—*Peoria Med. Monthly.*

**Chilblain Remedy.**—E. A. Rood sends us (National Druggist) the following, which he says is original with him, and never fails to give relief:

Take of—

| | | |
|---|---|---|
| ℞. | Sulphuric acid | 1 part, |
| | Turpentine | 8 parts. |

Mix in open vessel of glass or earthenware, pouring the acid in slowly and in small quantities. After heat subsides, bottle in two-ounce vials.

Sig. Apply at night, first washing the feet in hot water, and dry well. Apply this liniment, bathing in with heat, and put on stockings to prevent bed-stains.

**Wine of Pepsin.**—This (says the National Druggist) is No. 81 of the New York and Brooklyn Formulary :

| | |
|---|---|
| Pepsin, in scales | 128 grs. |
| Glycerin | 6 fl. drs. |
| Water | 1 fl. oz. |
| Hydrochloric acid | 75 min. |
| White wine, q. s. ad | 16 fl. ozs. |

Mix the water, glycerin, and hydrochloric acid, and agitate the pepsin with the mixture until it is dissolved ; then add enough white wine to make 16 fluid ounces ; allow to stand one week, then filter.

Each fluidram contains 1 grain of scaled pepsin.

*Remington's Wine of Pepsin.*—Take of—

| | |
|---|---|
| Saccharated pepsin | 1 Troy oz. |
| White wine | 14 fl. ozs. |
| Syrup | 2 fl. ozs. |

*Dubelle's Wine of Pepsin.*—Take of—

| | |
|---|---|
| Pepsin | 25 parts, |
| Orange flower water | 75 parts, |
| Alcohol | 50 parts, |
| White sugar | 150 parts, |
| Aromatic wine | 700 parts. |

Mix.

*Jensen's Wine of Pepsin.*—Take of—

| | |
|---|---|
| Crystal pepsin | 384 grs. |
| Tartaric acid | 10 grs. |
| Sherry wine | 13 fl. ozs. |
| Glycerin | 3 fl. ozs. |

Dissolve the tartaric acid, and then the pepsin, in the wine, and lastly add the glycerin. Filter for use.

Each fluidram contains 3 grains of pepsin.

**For Vomiting of Pregnancy.**—Wein (says the Medical World) extols the internal administration of cocaine for the arrest of the vomiting of pregnancy. The following is the formula :

| | | | |
|---|---|---|---|
| ℞. | Cocaine hydrochlorat | gr. xxx, | |
| | Aq. dest | ℥ j. | M. |

Sig. One teaspoonful every half hour until relieved.

**Chilblains.**—The Peoria Medical Monthly recommends—

| | | | |
|---|---|---|---|
| ℞. | Tincture iodi | 10 min. | |
| | Acidi salicylici | 10 gr. | |
| | Tincture benzoini comp | 1 oz. | M. |

Sig. Use externally.

